# Commentary: Switching to the Rubber Hand

**DOI:** 10.3389/fpsyg.2018.00588

**Published:** 2018-05-02

**Authors:** Andreas Kalckert

**Affiliations:** Psychology Section, University of Reading Malaysia, Iskandar Puteri, Malaysia

**Keywords:** body perception, sense of ownership, illusion, self, executive functions

The rubber hand illusion is a bodily illusion in which participants perceive a fake hand to be part of the body (Botvinick and Cohen, 1998). The illusion is basically a result of repeated stroking to both a model hand (in front of the participant) and the participant's real hand (hidden from view). It has been often anecdotally reported that the illusion occurs quite rapidly. However, more systematic investigations of the onset of the illusion are rare. The present study by Yeh and colleagues examines the relationship between executive functions like attention shifts and the experience of the rubber hand illusion, i.e., they investigate the role of top-down processes in the perception of the illusion (Yeh et al., 2017). Importantly, the authors use not only questionnaire ratings, but also measure the illusion onset, thus asking the participants when “…they begin to experience the rubber hand as belonging to the self…” (p.4). In the following I focus on this specific measurement as part of their investigation.

The authors report an illusion onset of approximately 60 s, so participants require about 60 s of visuotactile stimulation to experience the illusion. Similarly, a recent study by the same authors reported an average onset of approximately 100 s (Exp. 1) and 50 s (Exp. 2) (Lane et al., 2017). We can compare these results only to a few other studies that actually report the onset times. Ehrsson et al. (2004) reported an average onset of 11.3 s (SD 7.0s). In the study by Lloyd (2007) the average onset time was 5.6 s. However, it was defined as the onset of the referral of touch (Lloyd, 2007). The referral of touch reflects the experience of a fusion of the visual and tactile stimuli, and is one aspect of the experience in the rubber hand illusion. Aimola Davies et al. (2013) used a similar statement to measure the onset in the somatic and the visual tactile rubber hand illusion, and found an average of approximately 10 and 20 s respectively. Kalckert and Ehrsson (2017) measured the ownership onset time in the moving rubber hand illusion, a variant of the rubber hand illusion based on movements instead of visuotactile stimulation. They reported an average of approximately 22 s, for both active and passive movements. Thus, there is a relatively large difference between previously reported illusion onset times and the present finding.

In line with the authors observations these differences in illusion onset times could be explained by differences in executive functions across the mentioned studies. Alternatively, these differences could be rooted in a methodological aspect, i.e., in the way the illusion is induced and measured. The difference to the result by Lloyd (2007) and Aimola Davies et al. (2013) can be explained by the statement used to mark the illusion: here, participants were asked to indicate the occurrence of the referral-of-touch sensation and not ownership. The present study however, as well as the study by Ehrsson et al. (2004) and Kalckert and Ehrsson (2017), explicitly asked for the onset of the ownership experience, as the authors correctly point out (p. 4). When comparing onset times, it may be necessary to consider the frequency of the stimulation. Ehrsson and colleagues stimulated the hands at a rate of 1 Hz, so once every second. In the present study, the stimulation was applied at a rate of 0.5 Hz, thus at a slower pace. Therefore, there is a difference in the relative number of sensory events, i.e., correlated visual and tactile inputs, within a certain duration. An average onset of approximately 60 s with 0.5 Hz stimulation equates to 30 stimulations, which is more similar to approximately 11 sensory events (11 s with 1 Hz stimulation) or 22 sensory events (in case of the moving rubber hand illusion with 1 Hz finger taps). Thus, it may be equally important to take the relative frequency of the stimulation into account. The exact stimulation procedure can influence the onset times across studies.

As the authors state, the integrative processes underlying the illusion are determined by spatiotemporal factors of the visuotactile input, even when constrained by top-down processes. Indeed we know that timing of the stimuli and the distance between the hands are critical factors determining the illusion, factors reminiscent of the temporal and spatial rule in multisensory integration (Stein and Stanford, 2008; Ehrsson, 2012). It can be questioned, when using the onset measurement as a proxy of the illusion, if this onset is dependent on the actual distance of the hand. Spatial and temporal factors may interact in the generation of the illusion (see similar discussions in Zopf et al., 2010; Kalckert and Ehrsson, 2017). Most studies use a distance between 10 and 20 cm, and increasing the distance beyond approximately 30 cm typically abolishes the illusion (Lloyd, 2007; Preston, 2013; Kalckert and Ehrsson, 2014). The distance of the two hands is not known in this study, but the relative distance between the two hands may further affect the illusion onset as well as the illusion ratings in general, which could explain differences across studies.

As the authors highlight, the illusion onset measurement could be indeed a valuable additional measurement of the ownership illusion, complementing the more frequently used measurements like questionnaire ratings, proprioceptive drift, and skin conductance response. The present study adds to the rather sparse literature on the temporal dimension of the illusion. Illusion onset times allow to further characterize not only individual differences of participants, but more importantly the temporal dynamics of the illusion. The latter may be particular relevant from a practical or applied point of view. More applied research, which wants to generate sensations of ownership like in neuroprosthetics or virtual reality, has to take into account the relative onset of this experience. These may be influenced by executive functions. Both, processes of bottom-up perceptual input and top-down constraints determine when the participant will experience the illusion of having a new (rubber) hand as part of the own body.

## Author contributions

The author confirms being the sole contributor of this work and approved it for publication.

### Conflict of interest statement

The author declares that the research was conducted in the absence of any commercial or financial relationships that could be construed as a potential conflict of interest.

## References

[B1] Aimola Davies A. M. White R. C. Davies M. (2013). Spatial limits on the nonvisual self-touch illusion and the visual rubber hand illusion: Subjective experience of the illusion and proprioceptive drift. Conscious. Cogn. 22. 613-636. 10.1016/j.concog.2013.03.006 23644413

[B2] Botvinick M. Cohen J. (1998). Rubber hands “feel” touch that eyes see. Nature 391:756 10.1038/35784 9486643

[B3] Ehrsson H. H. (2012). The concept of body ownership and its relation to multisensory integration, in The New Handbook of Multisensory Processes, ed Stein B. E. (Cambridge, MA: MIT Press), 775–792.

[B4] Ehrsson H. H. Spence C. Passingham R. E. (2004). That's my hand! Activity in premotor cortex reflects feeling of ownership of a limb. Science 305, 875–877. 10.1126/science.1097011 15232072

[B5] Kalckert A. Ehrsson H. H. (2014). The spatial distance rule in the moving and classical rubber hand illusions. Conscious. Cogn. 30, 118–132. 10.1016/j.concog.2014.08.022 25286241

[B6] Kalckert A. Ehrsson H. H. (2017). The Onset Time of the Ownership Sensation in the Moving Rubber Hand Illusion. Front. Psychol. 8:344. 10.3389/fpsyg.2017.00344 28344566 PMC5345084

[B7] Lane T. Yeh S.-L. Tseng P. Chang A.-Y. (2017). Timing disownership experiences in the rubber hand illusion. Cogn. Res. Principl. Implic. 2:4. 10.1186/s41235-016-0041-4 28203632 PMC5281674

[B8] Lloyd D. M. (2007). Spatial limits on referred touch to an alien limb may reflect boundaries of visuo-tactile peripersonal space surrounding the hand. Brain Cogn. 64, 104–109. 10.1016/j.bandc.2006.09.013 17118503

[B9] Preston C. (2013). The role of distance from the body and distance from the real hand in ownership and disownership during the rubber hand illusion. ACTPSY 142, 177–183. 10.1016/j.actpsy.2012.12.005 23333877

[B10] Stein B. E. Stanford T. R. (2008). Multisensory integration: current issues from the perspective of the single neuron. Nat. Rev. Neurosci. 9, 255–266. 10.1038/nrn2331 18354398

[B11] Yeh S.-L. Lane T. J. Chang A.-Y. Chien S.-E. (2017). Switching to the Rubber Hand. Front. Psychol. 8:707. 10.3389/fpsyg.2017.02172 29312048 PMC5732964

[B12] Zopf R. Savage G. Williams M. A. (2010). Crossmodal congruency measures of lateral distance effects on the rubber hand illusion. Neuropsychologia 48, 713–725. 10.1016/j.neuropsychologia.2009.10.028 19913040

